# Supplementary figures and images for: Lipid bilayer properties potentially contributed to the evolutionary disappearance of betaine lipids in seed plants
Source: BMC Biol. 2023 Nov 28;21:275. doi: 10.1186/s12915-023-01775-z (PMC10685587; doi:10.1186/s12915-023-01775-z)

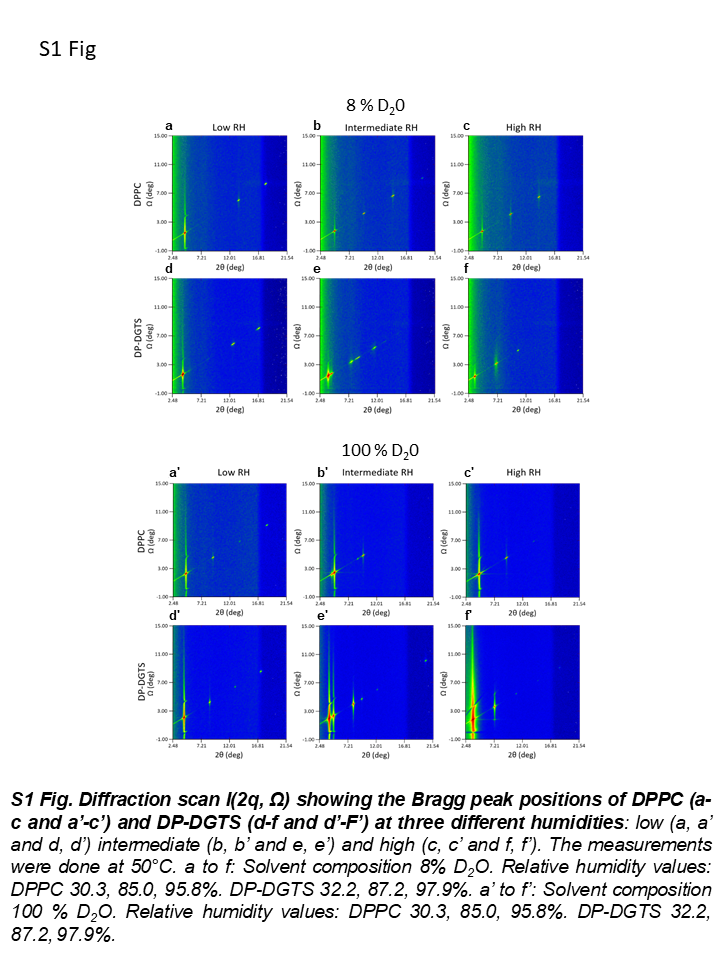

Supplement: Supplementary file 1 — Additional file 1: Figure S1. Diffraction scan I(2q, Ω) showing the Bragg peak positions of DPPC (a-c and’ a’-c’) and DP-DGTS (d-f and d’-f’) at three different humidities: low (a, a’ and d, d’) intermediate (b, b’ and e, e’) and high (c, c’ and f, f’). The measurements were done at 50°C. a to f: Solvent composition 8% D2O. Relative humidity values: DPPC 30.3, 85.0, 95.8%. DP-DGTS 32.2, 87.2, 97.9%. a’ to f’: Solvent composition 100 % D2O. Relative humidity values: DPPC 30.3, 85.0, 95.8%. DP-DGTS 32.2, 87.2, 97.9%. [file 12915_2023_1775_MOESM1_ESM.tif]

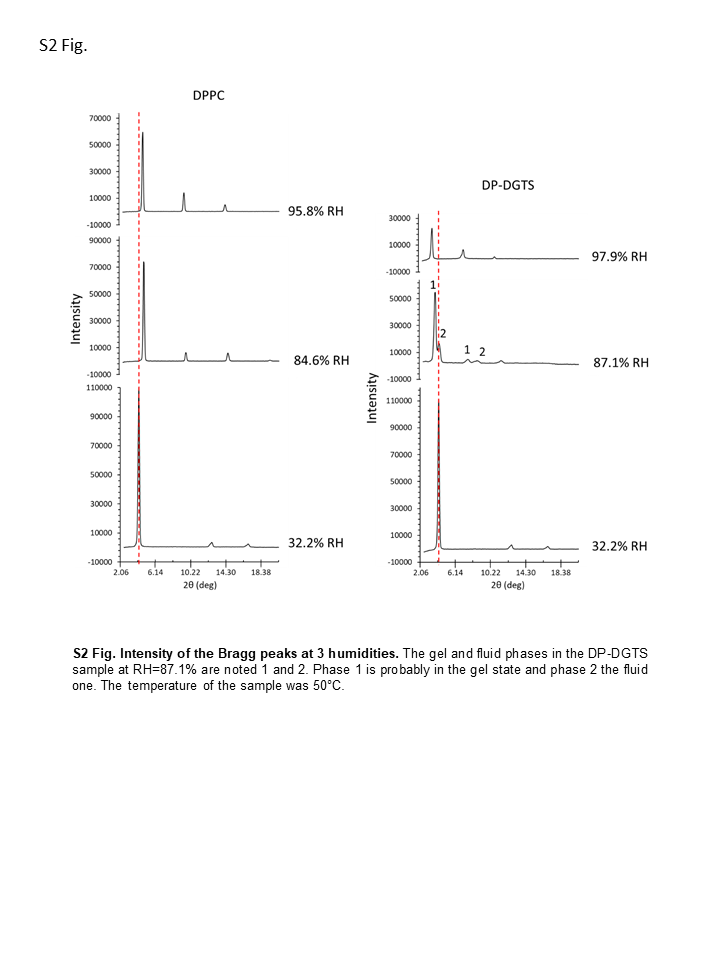

Supplement: Supplementary file 2 — Additional file 2: Figure S2. Intensity of the Bragg peaks at 3 humidities. The gel and fluid phases in the DP-DGTS sample at RH=87.1% are noted 1 and 2. Phase 1 is probably in the gel state and phase 2 the fluid one. The temperature of the sample was 50°C. [file 12915_2023_1775_MOESM2_ESM.tif]

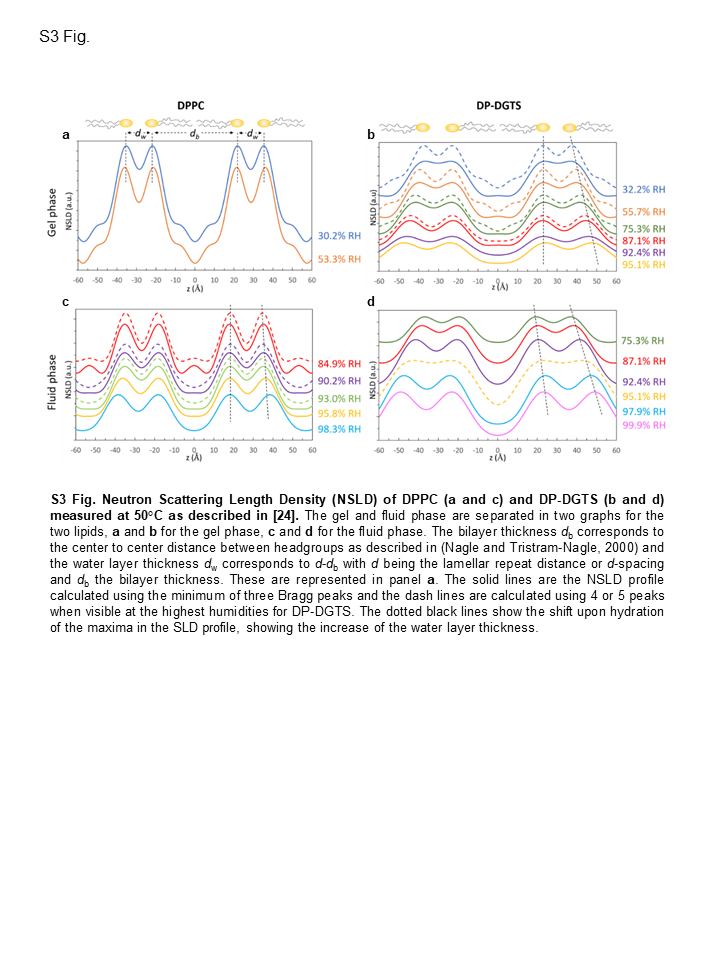

Supplement: Supplementary file 3 — Additional file 3: Figure S3. Neutron Scattering Length Density (NSLD) of DPPC (a and c) and DP-DGTS (b and d) measured at 50°C as described in [27]. The gel and fluid phase are separated in two graphs for the two lipids, a and b for the gel phase, c and d for the fluid phase. The bilayer thickness db corresponds to the center to center distance between headgroups as described in [29] and the water layer thickness dw corresponds to d-db with d being the lamellar repeat distance or d-spacing and db the bilayer thickness. These are represented in panel a. The solid lines are the NSLD profile calculated using the minimum of three Bragg peaks and the dash lines are calculated using 4 or 5 peaks when visible at the highest humidities for DP-DGTS. The dotted black lines show the shift upon hydration of the maxima in the SLD profile, showing the increase of the water layer thickness. [file 12915_2023_1775_MOESM3_ESM.tif]

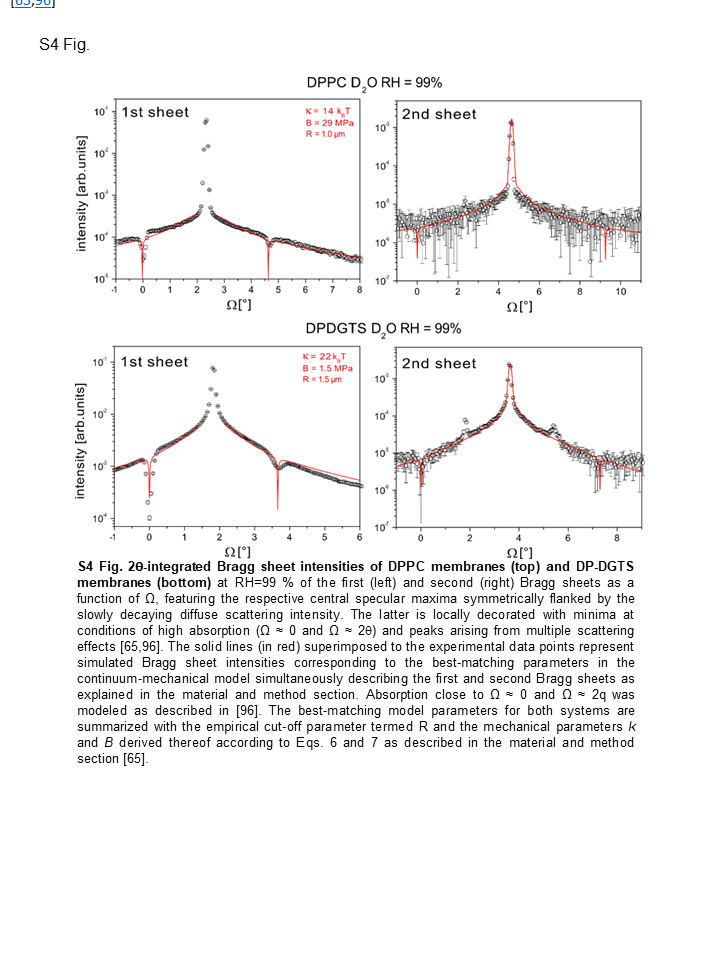

Supplement: Supplementary file 4 — Additional file 4: Figure S4. 2q-integrated Bragg sheet intensities of DPPC membranes (top) and DP-DGTS membranes (bottom) at RH=99 % of the first (left) and second (right) Bragg sheets as a function of Ω, featuring the respective central specular maxima symmetrically flanked by the slowly decaying diffuse scattering intensity. The latter is locally decorated with minima at conditions of high absorption (Ω ≈ 0 and Ω ≈ 2q) and peaks arising from multiple scattering effects [69, 71]. The solid lines (in red) superimposed to the experimental data points represent simulated Bragg sheet intensities corresponding to the best-matching parameters in the continuum-mechanical model simultaneously describing the first and second Bragg sheets as explained in the material and method section. Absorption close to Ω ≈ 0 and Ω ≈ 2q was modeled as described in [71]. The best-matching model parameters for both systems are summarized with the empirical cut-off parameter termed R and the mechanical parameters k and B derived thereof according to Eqs. 6 and 7 as described in the material and method section [69]. [file 12915_2023_1775_MOESM4_ESM.tif]

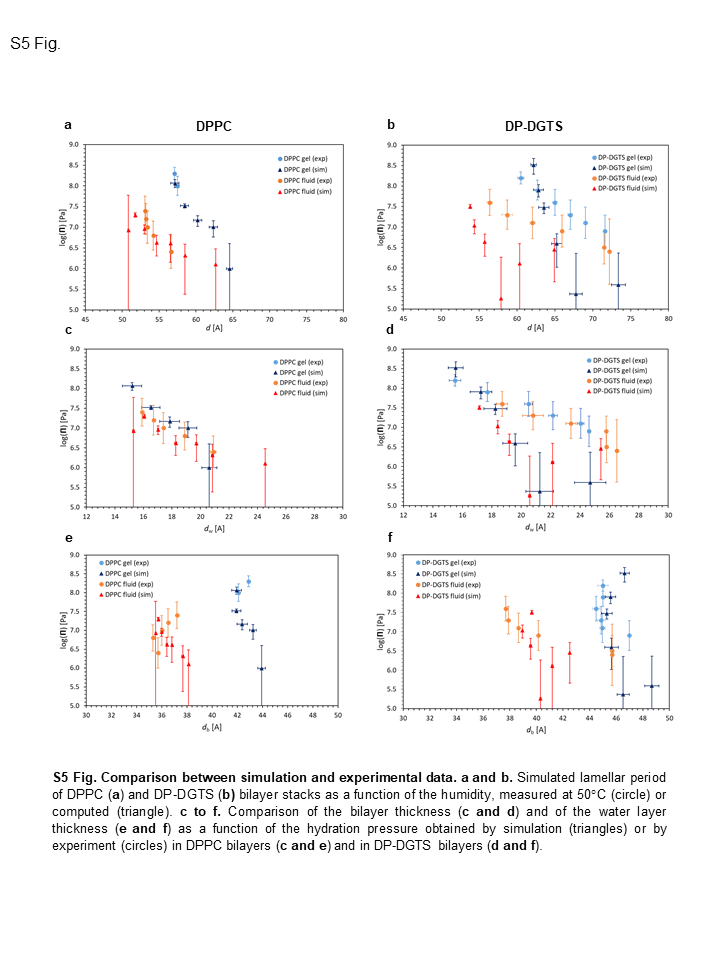

Supplement: Supplementary file 5 — Additional file 5: Figure S5. a and b. Simulated lamellar period of DPPC (a) and DP-DGTS (b) bilayer stacks as a function of the humidity, measured at 50°C (circle) or computed (triangle). c to f. Comparison of the bilayer thickness (c and d) and of the water layer thickness (e and f) as a function of the hydration pressure obtained by simulation (triangles) or by experiment (circles) in DPPC bilayers (c and e) and in DP-DGTS bilayers (d and f). [file 12915_2023_1775_MOESM5_ESM.tif]

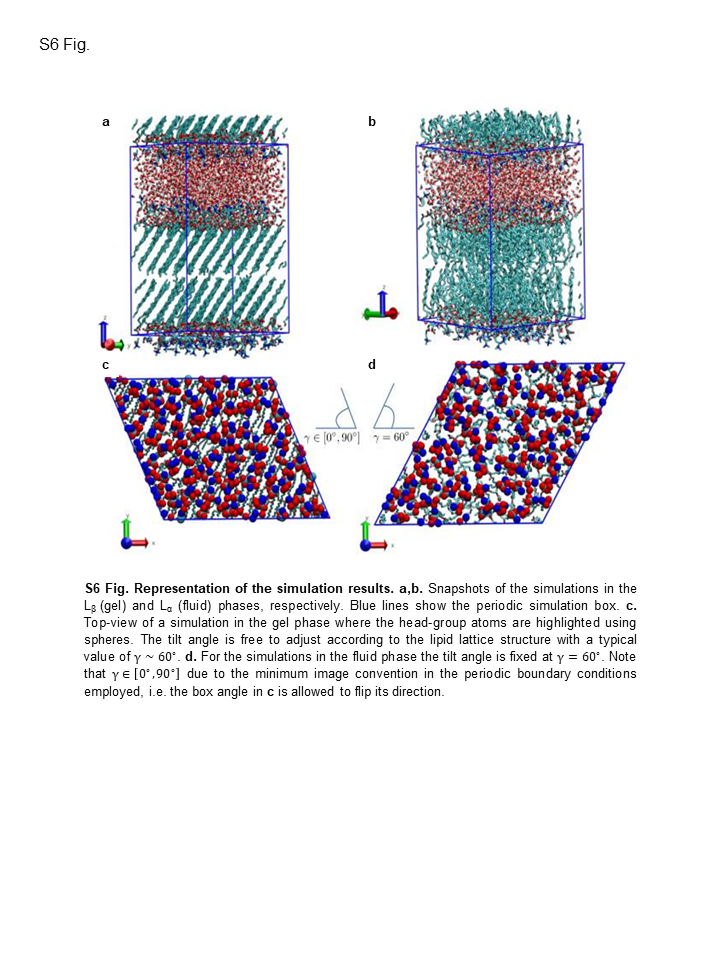

Supplement: Supplementary file 6 — Additional file 6: Figure S6. a,b. Snapshots of the simulations in the Lβ(gel) and Lα (fluid) phases, respectively. Blue lines show the periodic simulation box. c. Top-view of a simulation in the gel phase where the head-group atoms are highlighted using spheres. The tilt angle is free to adjust according to the lipid lattice structure with a typical value of . d. For the simulations in the fluid phase the tilt angle is fixed at . Note that due to the minimum image convention in the periodic boundary conditions employed, i.e., the box angle in C is allowed to flip its direction. [file 12915_2023_1775_MOESM6_ESM.tif]

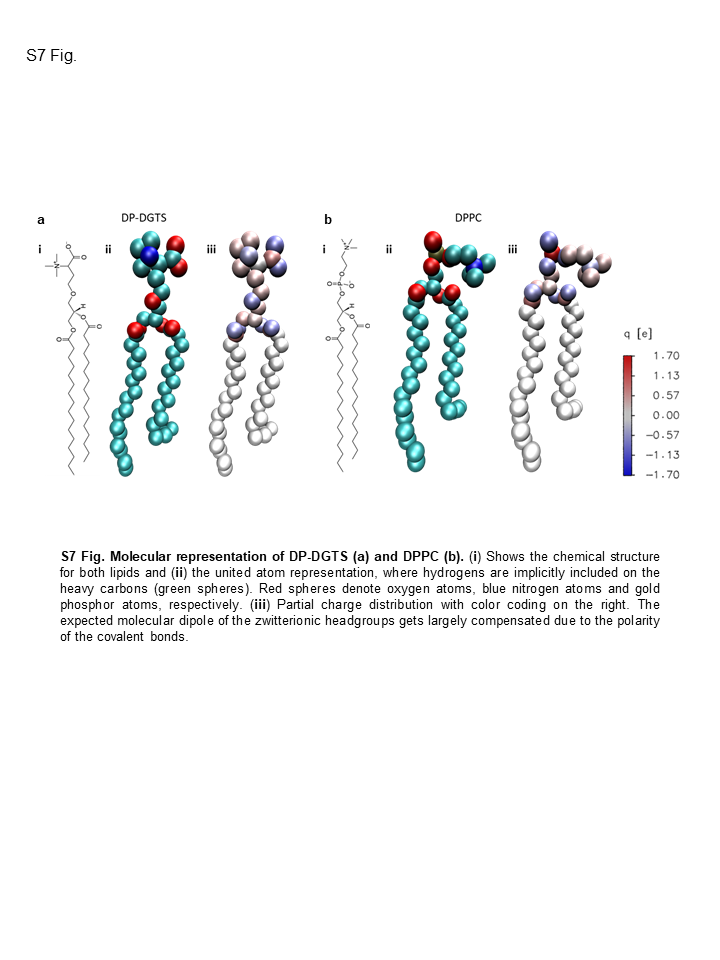

Supplement: Supplementary file 7 — Additional file 7: Figure S7. Molecular representation of DP-DGTS (a) and DPPC (b). (i) Shows the chemical structure for both lipids and (ii) the united atom representation, where hydrogens are implicitly included on the heavy carbons (green spheres). Red spheres denote oxygen atoms, blue nitrogen atoms and gold phosphor atoms, respectively. (iii) Partial charge distribution with color coding on the right. The expected molecular dipole of the zwitterionic headgroups gets largely compensated due to the polarity of the covalent bonds. [file 12915_2023_1775_MOESM7_ESM.tif]

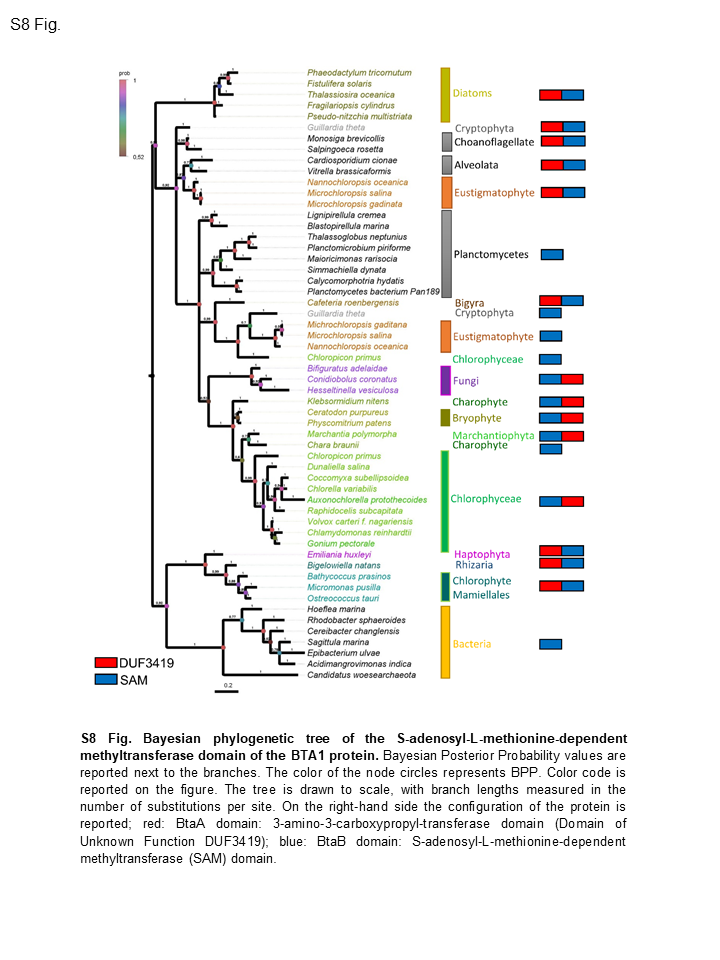

Supplement: Supplementary file 8 — Additional file 8: Figure S8. Bayesian phylogenetic tree of the S-adenosyl-L-methionine-dependent methyltransferase domain of the BTA1 protein. Bayesian Posterior Probability values are reported next to the branches. The color of the node circles represents BPP. Color code is reported on the figure. The tree is drawn to scale, with branch lengths measured in the number of substitutions per site. On the right-hand side the configuration of the protein is reported; red: BtaA domain: 3-amino-3-carboxypropyl-transferase domain (Domain of Unknown Function DUF3419); blue: BtaB domain: S-adenosyl-L-methionine-dependent methyltransferase (SAM) domain. [file 12915_2023_1775_MOESM8_ESM.tif]

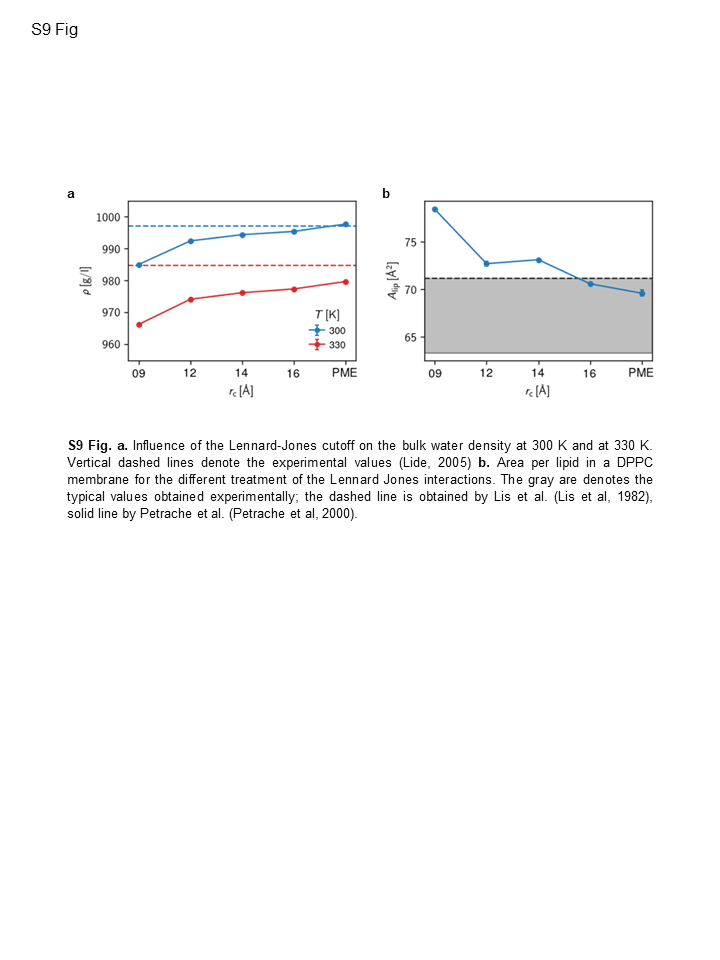

Supplement: Supplementary file 9 — Additional file 9: Figure S9. a. Influence of the Lennard-Jones cutoff on the bulk water density at 300 K and at 330 K. Vertical dashed lines denote the experimental values [81] b. Area per lipid in a DPPC membrane for the different treatment of the Lennard Jones interactions. The gray are denotes the typical values obtained experimentally; the dashed line is obtained by Lis et al. [82], solid line by Petrache et al. [83]. [file 12915_2023_1775_MOESM9_ESM.tif]
